# Full blood count as potential predictor of outcomes in patients undergoing cardiac resynchronization therapy

**DOI:** 10.1038/s41598-019-49659-z

**Published:** 2019-09-10

**Authors:** Nikolaos Papageorgiou, Debbie Falconer, Adam Ioannou, Tanakal Wongwarawipat, Sergio Barra, Dimitris Tousoulis, Wei Yao Lim, Fakhar Z. Khan, Syed Ahsan, Amal Muthumala, Ross J. Hunter, Malcolm Finlay, Antonio Creta, Edward Rowland, Martin Lowe, Oliver R. Segal, Richard J. Schilling, Pier D. Lambiase, Anthony W. Chow, Rui Providência

**Affiliations:** 1 Electrophysiology Department, Barts Heart Centre, St. Bartholomew’s Hospital, London, United Kingdom; 2 Institute of Cardiovascular Science, University College London, London, United Kingdom; 3 Cardiology Department, Papworth Hospital, Cambridge, United Kingdom; 4 1st Cardiology Department, Hippokration Hospital, Athens, Greece; 5 Institute of Health Informatics, University College London, London, United Kingdom

**Keywords:** Cardiac device therapy, Heart failure

## Abstract

Almost a third of patients fulfilling current guidelines criteria have suboptimal responses following cardiac resynchronization therapy (CRT). Circulating biomarkers may help identify these patients. We aimed to assess the predictive role of full blood count (FBC) parameters in prognosis of heart failure (HF) patients undergoing CRT device implantation. We enrolled 612 consecutive CRT patients and FBC was measured within 24 hours prior to implantation. The follow-up period was a median of 1652 days (IQR: 837–2612). The study endpoints were i) composite of all-cause mortality or transplant, and ii) reverse left ventricular (LV) remodeling. On multivariate analysis [hazard ratio (HR), 95% confidence interval (CI)] only red cell count (RCC) (p = 0.004), red cell distribution width (RDW) (p < 0.001), percentage of lymphocytes (p = 0.03) and platelet count (p < 0.001) predicted all-cause mortality. Interestingly, RDW (p = 0.004) and platelet count (p = 0.008) were independent predictors of reverse LV remodeling. This is the first powered single-centre study to demonstrate that RDW and platelet count are independent predictors of long-term all-cause mortality and/or heart transplant in CRT patients. Further studies, on the role of these parameters in enhancing patient selection for CRT implantation should be conducted to confirm our findings.

## Introduction

Cardiac resynchronization therapy (CRT) has emerged as an important alternative in treating heart failure (HF) patients with symptoms refractory to medical therapy^1^. Studies have shown that CRT induces reverse left ventricular remodeling in appropriately selected patients^2^, improves symptoms and reduces morbidity and mortality^3^.

Unfortunately, almost a third of patients do not respond favourably to CRT^4^. Several characteristics are associated with improved response, and thus survival following CRT implantation^5^. Optimization of patient selection for CRT will enable identification of non- responders, who might benefit from other treatment strategies.

Haemoglobin (Hb)^6^, mean platelet volume (MPV)^7^, mean corpuscular volume (MCV)^8^ and red cell distribution width (RDW)^9^ are associated with improved prognosis in HF, but their role in predicting outcomes in HF patients implanted with CRT remains unclear. These circulating biomarkers could potentially be useful tools in CRT patient selection, but their clinical use in that setting has not been sufficiently addressed. These tests are used in daily clinical practice and could be good candidates for this role as they are cheap and already routinely performed, avoiding the extra cost of expensive commercially available kits.

Previous studies^10,11^ attempted to examine the role of RDW in CRT response. However, these had short follow-up period, included small sample size, and did not provide conclusive evidence of an impact on survival through an association with reverse remodeling in the same cohort.

In the present study, we aimed to assess whether RDW could predict response (efficacy) after CRT implantation, in a large sample of HF patients and over a long follow-up period, including not only all-cause mortality and HF death or left ventricular (LV) remodeling but also other parameters related to CRT response.

Assessment of these endpoints could allow to determine whether such parameters are predictive of HF progression only, or whether these are also involved in the reverse remodeling process of CRT response.

## Materials and Methods

### Study patients

The study population consisted of 612 consecutive patients who were successfully implanted with a CRT at The Heart Hospital, University College of London (UCL) NHS Trust, London, United Kingdom (2000–2014). Cardiac resynchronization therapy devices with defibrillator (CRT-D) or pacemaker (CRT-P) were considered eligible. All participants gave written informed consent for the procedures, which was performed in accordance to the local and international guidelines (NICE/American College of Cardiology/American Heart Association/ European Society of Cardiology). There were no experiments involved in this retrospective study. All procedures (relevant protocol which was followed) were part of routine clinical practice according to the above guidelines, approved by the NHS as well as the UCLH/Heart Hospital institutional committee.

Briefly, in order for patients to undergo CRT implantation they had documented HF of New York Heart Association (NYHA) class II–IV symptoms despite optimal therapy, LV ejection fraction (LVEF) ≤35%, and QRS duration ≥120 ms, in line with the European Society of Cardiology (ESC) guidelines^1^. Choice of CRT-P or CRT-D was based on the patient’s clinical history, risk profile and history of arrhythmias. All variables at the time of the procedure and during follow-up were defined and categorized. Baseline data were collected on demographics, cardiac disease, echocardiographic results and medications (Table 1).

**Table 1:** Demographic characteristics of the study population.

Variables	Study population (N = 612)	Alive at follow-up (N = 247)	Death/ transplant (N = 332)	P
Age	65.1 ± 13.7	62.8 ± 13.2	66.9 ± 13.9	<0.001
Women N(%)	173 (28.4)	82 (33.2)	79 (23.8)	0.013
Diabetes N(%)	158 (26.0)	24.3 (60)	26.5 (88)	0.546
COPD N(%)	90 (14.8)	37 (15.0)	49 (14.8)	0.941
Previous stroke N(%)	40 (6.6)	11 (4.5)	32 (9.6)	0.019
Previous valve repair/replacement N(%)	51 (8.4)	19 (7.7)	26 (7.8)	0.951
AF N(%)	356 (58.5)	136 (55.1)	209 (63.1)	0.050
Peripheral vascular disease/ AAA N(%)	39 (6.4)	13 (4.2)	26 (6.8)	0.140
NYHA	2.7 ± 0.6	2.5 ± 0.6	2.9 ± 0.6	<0.001
Ischaemic CM N(%)	271 (44.5)	99 (40.1)	163 (49.1)	0.031
Secondary Prevention N (%)	87 (14.3)	37 (15.0)	47 (14.2)	0.781
eGFR	59 ± 22	64 ± 22	54 ± 20	<0.001
LVEF	29 ± 11	31 ± 12	27 ± 11	<0.001
QRS width	153 ± 33	155 ± 33	151 ± 33	0.232
CRT-D N(%)	465 (76.5)	194 (78.5)	260 (78.3)	0.947
CRT upgrade N(%)	182 (30.0)	66 (26.7)	93 (28.0)	0.731
Statin N(%)	326 (54.2)	134 (54.5)	179 (54.1)	0.925
Oral anticoagulants N(%)	261 (43.3)	112 (45.5)	137 (41.7)	0.358
Antiplatelets N(%)	271 (45.0)	108 (43.9)	152 (45.8)	0.653
Beta-blockers N(%)	407 (67.6)	174 (70.7)	212 (64.0)	0.092
ACEi/ARB-II N(%)	521 (86.5)	216 (87.8)	283 (85.5)	0.423
Spironolactone N(%)	349 (58.1)	140 (56.9)	193 (58.3)	0.737
Loop diuretic N(%)	465 (77.4)	161 (65.4)	285 (86.1)	<0.001
Haemoglobin	12.7 ± 1.8	129 ± 17	124 ± 19	0.001
Red cell count	4.3 ± 0.6	4.4 ± 0.6	4.2 ± 0.6	0.012
Mean corpuscular volume	89.0 ± 7.5	88.8 ± 6.3	89.1 ± 8.3	0.648
Red Cell Distribution Width	14.8 ± 2.0	14.2 ± 1.5	15.3 ± 2.1	<0.001
White Blood Cell count	7.8 ± 3.3	7.7 ± 2.6	10.5 ± 4.3	0.310
Neutrophiles	65 ± 11	63 ± 11	67 ± 11	<0.001
Lymphocytes	23 ± 10	25 ± 9	21 ± 10	<0.001
Neutrophile/Lymphocyte ratio	3.8 ± 3.4	3.3 ± 3.5	4.2 ± 3.4	0.001
Platelets	219 ± 77	232 ± 84	210 ± 71	<0.001
Mean Platelet volume	11.2 ± 4.5	11.0 ± 1.1	11.5 ± 5.9	0.180

Abbreviations. NYHA: New York Heart Association; ACE-I: angiotensin converting enzyme inhibitors; ARB-II: Angiotensin II receptor blockers; CRT-D; cardiac resyncronization therapy-defibrillator; CRT: cardiac resynchronization therapy; eGFR: estimated glomerular filtration rate; CM: cardiomyopathy; LVEF: left ventricular ejection fraction; COPD: chronic obstructive pulmonary disease; AF: atrial fibrillation; AAA: abdominal aortic aneurysm; HR: hazard ratio; CI: confidence of interval. Neutrophiles and Lympocytes are %. Values are present as mean ± SD.

Exclusion criteria were: age younger than 18 years, requirement of intravenous inotropic drug therapy or having an estimated life expectancy of less than 12 months due to comorbidities other than heart failure as previously described^12^.

### Measurement of full blood count parameters (FBC)

Blood samples were obtained from all patients less than 24 hours before the procedure. Ethylenediamine tetraacetic acid (EDTA) samples were processed by our laboratory (University College London Hospital) for full blood count parameters including Hb, white cell count (WCC), differential count [percentage (%) of neutrophils, (%) of lymphocytes, (%) of monocytes, (%) of eosinophils and (%) of basophils]^4^, platelet count, red cell count (RCC), RDW and MCV with commercially available methods (fluorescent flow cytometry, Sysmex XE-2100).

### Device programming

Devices were programmed with two ventricular tachycardia zones *ab initium*, according to the patient’s age and previously documented ventricular arrhythmias, as some patients had CRT implanted before the MADIT-RIT trial^13^. Anti-tachycardia pacing (ATP) and shocks were programmed in the ventricular tachycardia (VT) and ventricular fibrillation (VF) zone. VT detection was programmed starting at 170bpm if no previous documentation of slow VT was present. Detection time was set between 2.5 s and 9.0 s (according to the manufacturer) in the VT zone and between 1.0 s and 5.0 s in the VF zone. High-rate timeout was turned off and the supraventricular tachycardia discriminator algorithms were switched on. Adjustment of therapies and detection zones was performed during follow-up or following documented events of arrhythmia^12^. After 2012 some patients had quadripolar LV leads implanted.

### Follow-up period, primary and secondary endpoints

The patients were followed-up for a median period of 1652 days post-CRT implantation. The data were collected retrospectively through hospital electronic records, while additional information, where needed, was retrieved from paper notes. Data from our local clinic records and stored device electrograms (EGMs) regarding episodes of tachycardia and related therapies or inappropriate shocks delivered were also collected. The latter were analysed by Senior Physiologists with expertise in cardiac electrophysiology and a Consultant Electrophysiologist/ Senior Electrophysiology Fellow. Episodes of sustained VT terminated through ICD intervention were logged. Effective ATP was defined as overdrive ventricular pacing able to restore sinus rhythm during VT or VF episodes. Appropriate shocks were reported when shock therapy terminated VT or VF. The presence of either an appropriate/effective shock or ATP was classified as appropriate ICD intervention. Inappropriate shocks, delivered due to mis-classification of supraventricular tachycardia, sinus tachycardia, fast conducted atrial fibrillation or artefact were also assessed^12^.

The study endpoints were defined as: (i) all-cause mortality/heart transplant and (ii) reverse LV remodelling.

### Statistical analysis

PASW Statistics (SPSS Inc, Chicago, IL) version 18.0 was used for descriptive and inferential statistical analysis.

Comparison of continuous variables was performed using the ANOVA test, or its non-parametric equivalent, when appropriate. Homogeneity of variance was checked using the Levene’s test. Ratios were compared using the Chi-square test, or the Fisher’s test when appropriate. Analysis of time-to-event data was done through Cox regression. Univariate and multivariate Cox regression (using the forward likelihood ratio method, with a probability for step-wise of 0.05) was performed for identifying independent predictors of all-cause mortality or heart transplant.

Cut-off points for FBC variables which associated with all-cause mortality or heart transplant, were defined based on tertiles as these allowed division of the cohort into three groups with a nearly identical number of patients in each. This allowed sufficient power for comparisons showing a 50% difference among groups for endpoints occurring in at least 25% of patients (β = 0.8, and α = 0.05).

Kaplan-Meier curves were used to illustrate the association of different biomarker tertiles with the study endpoints, and the log rank test was used to assess for the presence of differences. Results with *P* < 0.05 were regarded as significant.

## Results

In the present study, we enrolled 612 consecutive HF patients in whom a CRT was implanted and FBC was measured within 24 hours prior to implantation.

The follow-up period was a median of 1,652 days (IQR: 837–2,612). Overall, all-cause mortality or cardiac transplant occurred in 56.3% of patients, while LVEF improvement of greater than 5% was seen in 55.8% of patients.

### Predictors of all-cause mortality/transplant

Cox proportional hazards univariate model revealed that besides established predictive factors [age, gender, previous stroke, NYHA, ischaemic substrate, estimated glomerular filtration rate (eGFR), LVEF], Hb, RCC, RDW, neutrophils, lymphocytes, neutrophile/lymphocyte ratio, platelet count and MPV were predictors of all-cause mortality/transplant (Table 2).

**Table 2:** Predictors of all-cause mortality.

Variable	Univariate			Multivariate		
	HR	95% CI	P	HR	95% CI	P
Age	1.02	1.01–1.03	<0.001	—	—	—
Gender	0.71	0.56–0.89	0.003	0.73	0.56–0.95	0.018
Diabetes	1.15	0.91–1.44	0.24	—	—	—
COPD	0.91	0.69–1.20	0.49	—	—	—
Previous stroke	1.49	1.04–2.13	0.03	—	—	—
Previous valve repair/replacement	1.10	0.77–1.57	0.61	—	—	—
AF	1.13	0.92–1.49	0.26	—	—	—
Peripheral vascular disease/ AAA	1.32	0.88–1.96	0.18	—	—	—
NYHA	2.13	1.79–2.53	<0.001	1.63	1.33–1.99	<0.001
Ischaemic CM	1.39	1.14–1.70	0.001	—	—	—
Secondary Prevention	0.95	0.71–1.28	0.75	—	—	—
eGFR	0.98	0.98–0.99	<0.001	0.99	0.99–1.00	0.05
LVEF	0.98	0.97–0.99	<0.001	0.99	0.98–1.00	0.04
QRS width	1.00	1.00–1.00	0.37	—	—	—
CRT-D	0.84	0.56–1.07	0.16	—	—	—
CRT upgrade	1.08	0.87–1.34	0.50	—	—	—
Statin	1.11	0.90–1.35	0.33	—	—	—
Oral anticoagulants	0.91	0.74–1.12	0.38	—	—	—
Antiplatelets	1.02	0.83–1.24	0.87	—	—	—
Beta-blockers	0.89	0.72–1.10	0.27	—	—	—
ACEi/ARB-II	1.05	0.80–1.39	0.72	—	—	—
Spironolactone	1.12	0.91–1.37	0.28	—	—	—
Loop diuretic	2.30	1.73–3.06	<0.001	1.93	1.40–2.66	<0.001
Haemoglobin	0.99	0.98–0.99	<0.001	—	—	—
Red cell count	0.72	0.60–0.86	<0.001	0.74	0.61–0.91	0.004
Mean corpuscular volume	1.00	0.99–1.02	0.85	—	—	—
Red Cell Distribution Width*	1.22	1.17–1.27	<0.001	1.19	1.13–1.26	<0.001
White Blood Cell count*	1.01	0.97–1.04	0.77	—	—	—
Neutrophiles (%)	1.03	1.02–1.04	<0.001	—	—	—
Lymphocytes (%)	0.96	0.95–0.98	<0.001	0.99	0.99–1.00	0.03
Neutrophile/Lymphocyte ratio	1.04	1.02–1.07	<0.001	—	—	—
Platelets*	0.997	0.996–0.999	<0.001	0.99	0.99–1.00	<0.001
Mean Platelet volume	1.02	1.01–1.04	0.006	—	—	—

Abbreviations. NYHA: New York Heart Association; ACE-I: angiotensin converting enzyme inhibitors; ARB-II: Angiotensin II receptor blockers; CRT-D; cardiac resyncronization therapy-defibrillator; CRT: cardiac resynchronization therapy; eGFR: estimated glomerular filtration rate; CM: cardiomyopathy; LVEF: left ventricular ejection fraction; COPD: chronic obstructive pulmonary disease; AF: atrial fibrillation; AAA: abdominal aortic aneurysm; HR: hazard ratio; CI: confidence of interval.

*RDW (per % increase); *platelets (per % increase); *white cell count (per % increase).

However, on multivariate analysis [hazard ratio (HR), 95% confidence interval (CI)] of the FBC parameters only RCC [0.74(0.61–0.91), p = 0.004], RDW [1.19(1.13–1.26), p < 0.001], percentage of lymphocytes [0.99(0.99–1.00), p = 0.03] and platelet count [0.99(0.99–1.00), p < 0.001] remained independent predictors (Tables 2 and S1).

### Predictors of reverse LV remodeling

When we examined for FBC predictors of LV remodeling, RDW [0.92(0.88–0.97), p = 0.004] and platelet count [1.01(1.00–1.01), p = 0.008] remained independent predictors in multivariate Analysis (Table 3).

**Table 3:** Predictors of reverse LV remodelling (LVEF 5% improvement).

Variable	Univariate			Multivariate		
	OR	95% CI	P	OR	95% CI	P
Age	1.01	1.00–1.01	0.001	—	—	—
Gender	1.44	1.08–1.93	0.01	—	—	—
Diabetes	1.29	0.93–1.78	0.12	—	—	—
COPD	1.27	0.84–1.91	0.26	—	—	—
Previous stroke	1.86	0.97–3.56	0.06	—	—	—
Previous valve repair/replacement	1.04	0.60–1.80	0.90	—	—	—
AF	1.18	0.96–1.45	0.18	—	—	—
Peripheral vascular disease/AAA	1.54	0.77–3.09	0.23	—	—	—
NYHA	1.09	1.03–1.16	0.003	—	—	—
Ischaemic CM	1.32	0.89–1.45	0.32	—	—	—
Secondary Prevention	1.16	0.75–1.80	0.50	—	—	—
eGFR	1.01	1.00–1.01	<0.001	—	—	—
LVEF	1.01	1.00–1.01	0.004	—	—	—
QRS width	1.01	1.00–1.01	0.001	1.01	1.00–1.01	0.05
CRT-D	1.26	1.05–1.51	0.015	—	—	—
CRT upgrade	1.08	0.87–1.34	0.50	—	—	—
Statin	1.41	0.13–1.76	0.003	—	—	—
Oral anticoagulants	1.22	0.95–1.55	0.11	—	—	—
Antiplatelets	1.41	1.11–1.81	0.006	—	—	—
Beta-blockers	1.39	1.14–1.70	0.001	—	—	—
ACEi/ARB-II	1.36	1.14–1.62	0.001	—	—	—
Spironolactone	1.24	1.00–1.54	0.046	—	—	—
Loop diuretic	1.34	1.12–1.61	0.002	—	—	—
Haemoglobin	1.01	1.00–1.01	0.003	—	—	—
Red cell count	1.06	1.02–1.10	0.006	—	—	—
Mean corpuscular volume	1.01	1.00–1.01	0.004	—	—	—
Red Cell Distribution Width*	0.99	0.99–1.00	0.02	—	—	—
White Blood Cell count*	1.00	0.99–1.00	0.83	—	—	—
Neutrophiles (%)	1.01	1.00–1.01	0.009	—	—	—
Lymphocytes (%)	1.01	1.00–1.02	0.005	—	—	—
Neutrophile/Lymphocyte ratio	1.04	1.00–1.08	0.03	—	—	—
Platelets*	1.01	1.00–1.01	0.002	1.01	1.00–1.01	0.008
Mean Platelet volume	1.02	1.01–1.04	0.003	—	—	—

Abbreviations. NYHA: New York Heart Association; ACE-I: angiotensin converting enzyme inhibitors; ARB-II: Angiotensin II receptor blockers; CRT-D; cardiac resyncronization therapy-defibrillator; CRT: cardiac resynchronization therapy; eGFR: estimated glomerular filtration rate; CM: cardiomyopathy; LVEF: left ventricular ejection fraction; COPD: chronic obstructive pulmonary disease; AF: atrial fibrillation; AAA: abdominal aortic aneurysm; HR: hazard ratio; CI: confidence of interval.

*RDW (per % increase); *platelets (per % increase); *white cell count (per % increase).

### Red cell distribution width, platelet count and neutrophils (%) association with endpoints

Given that RDW, platelet count and neutrophils were found to be independent predictors of the pre-specified endpoints, further analyses were performed to assess whether different levels of those predictors could have a different impact on patients’ prognosis/events. We therefore examined the CRT-related mortality/events within tertiles of the abovementioned parameters.

**Figure 1:**
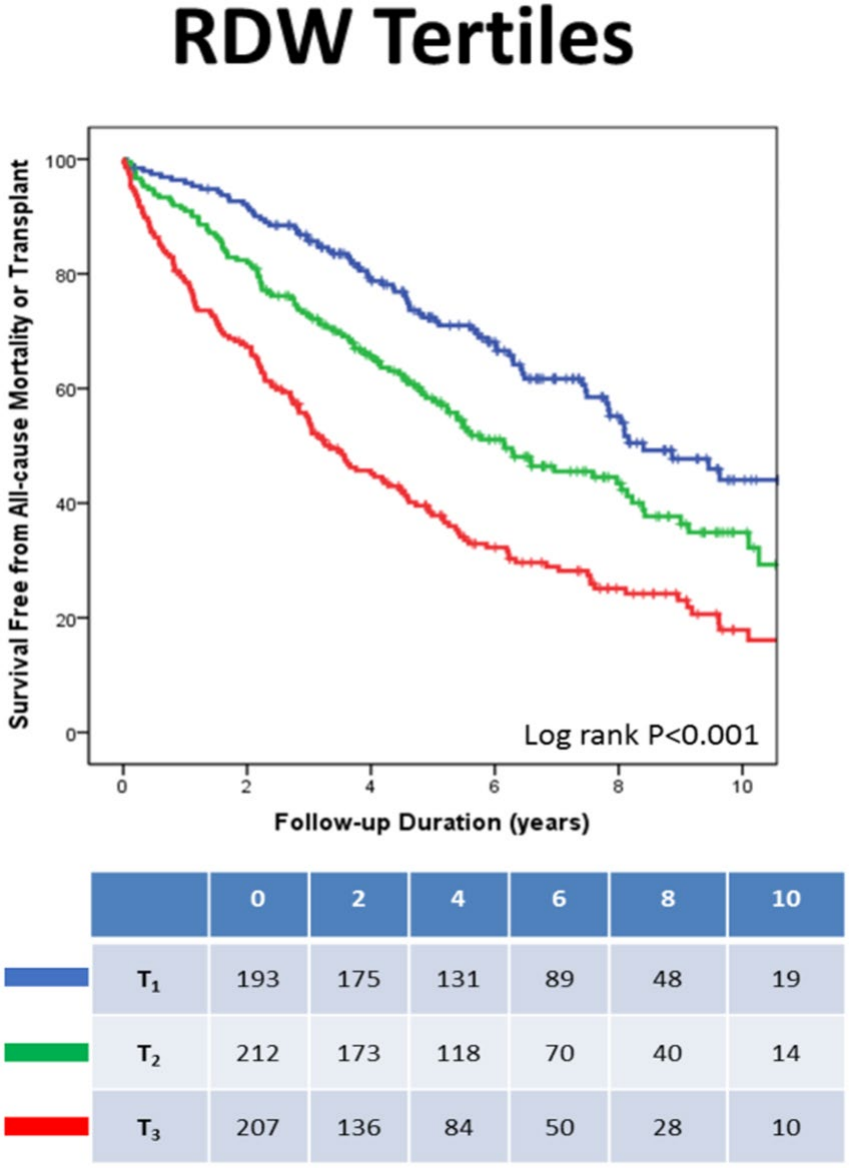
RDW tertiles and all-cause mortality/transplant. *RDW tertiles: <13.7, 13.7 to 15.1 and > = 15.1.

**Figure 2:**
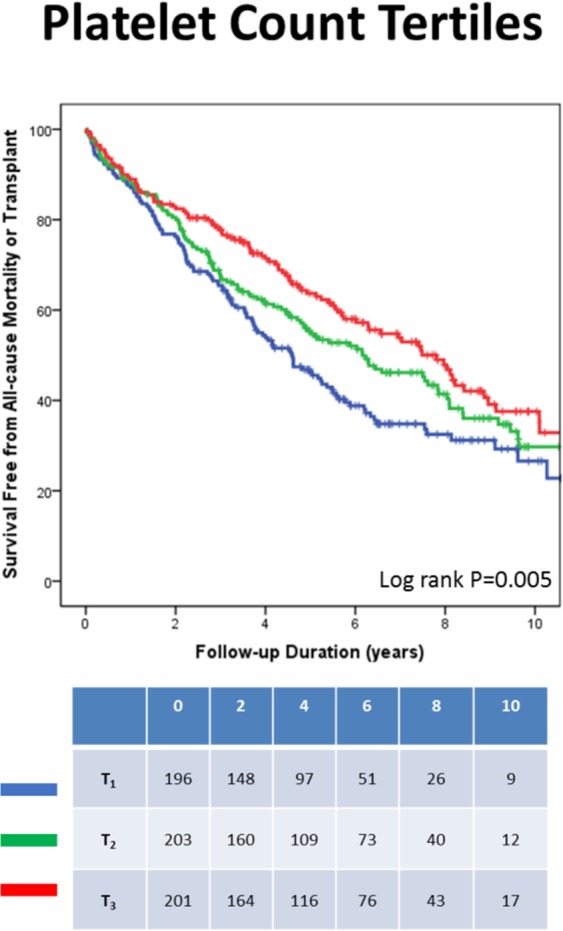
Platelet count tertiles and all-cause mortality/transplant. **Platelets tertiles: <183, 183 to 236 and > = 236.

During the follow-up period, patients in the lower RDW tertiles had significantly better survival free from all-cause mortality/transplant (Fig. 1). Similarly, patients in the higher platelet count tertiles had also significantly better survival free from all-cause mortality/transplant (Fig. 2).

## Discussion

In the present study we observed that full blood count parameters measured within 24 hours prior to CRT implantation may have a role in predicting mortality and relevant events in HF patients. Interestingly, the same parameters predicting survival are also predictors of LV reverse remodelling, suggesting that a potential involvement in the CRT response process may explain a survival benefit. Among the FBC parameters measured, only RDW, platelet count and percentage of lymphocytes were found to independently predict the endpoints of the study.

Previous studies have shown that HF patients with higher RDW may have worse prognosis than those with lower RDW. However, high RDW is associated with increased all-cause mortality in acute HF patients with preserved LVEF, but not in patients with reduced LVEF^14^. A potential effect of RDW on reverse remodelling has been previously suggested in a small study which included only a third of our cohort size and reported that patients in the highest RDW quartile experienced significantly less improvement in LVEF^10^. To date no study has reported that RDW and platelet count are associated with improved survival, and this survival benefit is potentially related to their effect on CRT response and their impact on heart failure death/transplant (Table S1). This suggests that there is a link which goes beyond the association between RDW and overall frailty or all-cause mortality and may be able to predict HF-related outcomes. Lower RDW and higher platelet count can predict both reverse remodelling and survival of patients who had a CRT implanted. We therefore, have two parameters, clinical and echocardiographic implicating a possible involvement of the examined parameters in the process of the CRT response.

The exact mechanisms underlying the association between RDW and poor prognosis of patients with HF is still unclear. It has been hypothesized that this association is mediated by inflammation, as the latter inhibits erythrocyte maturation and promotes the migration of reticulocytes into the peripheral circulation, thereby increasing RDW^15^. In addition to this, RDW has been correlated with inflammatory markers^16^.

The role of baseline RDW and its serial changes after CRT implant remains unknown. In a cohort of 148 patients RDW levels were measured before and 3 months after CRT implant^17^. It was found that increased and stable-high RDW levels were associated with both LV remodelling and outcome after CRT. However, RDW did not have any incremental predictive role^17^. Moreover, Topaz *et al*.^18^ measured RDW at 3 time points before and post-CRT implant in a sample of 156 patients. In a 61 month follow up-period, they reported that elevated RDW levels before and post-CRT implantation are associated with all-cause mortality.

Previous small underpowered studies, with very short follow up period, attempted to assess the predictive role of RDW in patients undergoing CRT^11^. An elevated RDW level before and after CRT implantation was independently associated with all-cause mortality. Moreover, increased RDW levels predict long-term mortality of CRT patients independently of the N-terminal prohormone of brain natriuretic peptide (NT-proBNP)^19^.

Apart from RDW, MPV has also been associated with HF, as well as CRT outcomes^7^. In our study, we found that although MPV could be a predictor of the endpoints, this did not remain the case in a multivariate model of analysis.

Further research on the predictive role of the FBC has shown that relative neutrophilia on admission to hospital in patients with acute myocardial infarction is significantly associated with the early development of congestive HF^20^. Moreover, the neutrophil/lymphocyte ratio is elevated in chronic heart failure and predicts outcomes after CRT implantation^21^. Our analysis showed that the neutrophil/lymphocyte ratio could predict all-cause mortality/transplant as well as HF death/transplant and LV remodelling. However, this ratio was less predictive of these endpoints on multivariate analysis.

There is ongoing debate as to whether inflammation plays a role in the prognosis of patients with HF. The results of the RENEWAL study were sufficiently unfavorable as to rule out a clinically relevant benefit of targeted anti-cytokine therapy with the soluble tumor factor necrosis antagonist Etanercept on the rate of death or HF hospitalization in HF^22^. On the contrary, the recently published CANTOS trial^23^ showed that canakinumab, a monoclonal antibody targeting interleukin-1β led to a significantly lower rate of recurrent cardiovascular events than placebo. Even though this study included a population different from ours, it suggested that anti-inflammatory drugs may have an impact in patients with cardiovascular disease. These are interesting findings at the light of our observation of the association of % lymphocyte with survival.

The analysis of molecular pathways involved in the mediation of the association of these markers with improved survival is of great importance. A better understanding of this could potentially lead to the identification of druggable targets and mediators involved in the process, which could thereby lead to the development of new drugs with a positive impact on prognosis/survival. Our study demonstrated that FBC parameters associated with red blood cells and platelets could predict mortality/events in HF patients undergoing CRT. Therefore, we can hypothesize that these may mediate different pathways, rather than inflammation alone as it is widely speculated. This is well supported by the fact that they are both independent prognostic factors.

Most importantly, our study parameters are routinely available in clinical practice as part of a routine full blood count and biochemistry measured during pre-operative assessment, making them inexpensive tests with a good prognostic value.

### Limitations

We acknowledge the fact that C-reactive protein (CRP) and brain natriuretic peptide (BNP)/NT-proBNP levels were not available for most of the patients. Therefore, we are not able to link our findings with inflammation and other neuro-humoral pathways. In addition, we would like to mention the temporal variation of RDW or other parameters which may be of interest and should be further assessed by future studies. Unfortunately, as most of our patients were followed up in other district centers or didn’t have those parameters routinely repeated during their follow-up, it would have not been easy to answer such a question. Either way post-device implant those parameters are not usually-strictly followed-up unless this is strongly indicated. We also believe that more complete information would allow better interpretation of our data, although the percentage of missing data was comparable across the study groups. Finally, a multi-center study could provide more accurate and valid results, however this study had the power to highlight significant associations and differences. To the best of our knowledge this is the largest single center evaluation of the role of full blood count parameters in HF patients undergoing CRT with long term follow-up.

## Conclusions

In this large cohort of heart failure patients implanted with CRT, we have found that RCC and RDW, percentage of lymphocytes and platelet count predicted all-cause mortality. Of these parameters, only RDW and platelet count were also independent predictors of reverse LV remodelling, suggesting that these could potentially participate in the CRT response process.

Our findings suggest that RDW and platelet count could enhance the patient selection process for CRT implantation by aiding physicians with risk stratification and estimation of survival benefit.

## Supplementary information

Table S1

## Data Availability

We comply with Scientific reports data availability policy and we will make any data available to reviewers or referees if needed.
