# Supplementary material for: Full blood count as potential predictor of outcomes in patients undergoing cardiac resynchronization therapy
Source: Sci Rep. 2019 Sep 10;9:13016. doi: 10.1038/s41598-019-49659-z (PMC6736835; doi:10.1038/s41598-019-49659-z)
Supplement: Supplementary file 1 — Table S1 [file 41598_2019_49659_MOESM1_ESM.docx]

**Full blood count as potential predictor of outcomes in patients undergoing**

**cardiac resynchronization therapy**

Nikolaos Papageorgiou^1,2*^, Debbie Falconer^2*^, Adam Ioannou^2^, Tanakal Wongwarawipat^2^, Sergio Barra^3^, Dimitris Tousoulis^4^, Wei Yao Lim^1^, Fakhar Z. Khan, Syed Ahsan^1^, Amal Muthumala^1^, Ross J. Hunter^1^, Malcolm Finlay^1^, Antonio Creta^1^, Edward Rowland^1^, Martin Lowe^1^, Oliver R. Segal^1^, Richard J. Schilling^1^, Pier D. Lambiase^1, 2^, Anthony W. Chow^1^, Rui Providência^1, 2, 5^

**equally contributed*

1. Barts Heart Centre, St. Bartholomew’s Hospital, London, United Kingdom
2. University College London, London, United Kingdom
3. Cardiology Department, Papworth Hospital, United Kingdom
4. 1^st^ Cardiology Department, Hippokration Hospital, Athens, Greece
5. Institute of Health Informatics, University College London, London, United Kingdom

**Running title:** FBC and cardiac resynchronization therapy

**Word count:** 2808 (Abstract & manuscript)

**Corresponding author:**

**Dr Nikolaos Papageorgiou MD PhD FESC**

Barts Heart Centre, St. Bartholomew’s Hospital

West Smithfield, EC1A 7BE

London, United Kingdom

Email: [drnpapageorgiou@yahoo.com](mailto:drnpapageorgiou@yahoo.com)

**Table S1**. Predictors of heart failure death/transplant.

|  | Univariate | | | Multivariate | | |
| --- | --- | --- | --- | --- | --- | --- |
| Variable | HR | 95%CI | P | HR | 95%CI | P |
| Age | 1.00 | 0.99-1.01 | 0.67 | - | - | - |
| Gender | 0.71 | 0.51-1.00 | 0.05 | - | - | - |
| Diabetes | 1.12 | 0.80-1.57 | 0.51 | - | - | - |
| COPD | 0.81 | 0.52-1.25 | 0.34 | - | - | - |
| Previous stroke | 1.16 | 0.63-2.14 | 0.63 | - | - | - |
| Previous valve repair/replacement | 1.11 | 0.65-1.89 | 0.70 | - | - | - |
| AF | 1.34 | 0.97-1.84 | 0.07 | - | - | - |
| Peripheral vascular disease/ AAA | 0.71 | 0.31-1.60 | 0.40 | - | - | - |
| NYHA | 2.82 | 2.16-3.67 | <0.001 | 1.95 | 1.46-2.60 | <0.001 |
| Ischaemic CM | 0.98 | 0.72-1.33 | 0.90 | - | - | - |
| Secondary Prevention | 0.69 | 0.42-1.14 | 0.14 | - | - | - |
| eGFR | 0.98 | 0.97-0.99 | <0.001 | 0.98 | 0.98-0.99 | 0.001 |
| LVEF | 0.97 | 0.96-0.99 | <0.001 | - | - | - |
| QRS width | 0.99 | 0.99-1.00 | 0.003 | 0.99 | 0.99-1.00 | 0.004 |
| CRT-D | 1.09 | 0.74-1.59 | 0.67 | - | - | - |
| CRT upgrade | 1.15 | 0.84-1.59 | 0.39 | - | - | - |
| Statin | 0.82 | 0.61-1.11 | 0.20 | - | - | - |
| Oral anticoagulants | 1.04 | 0.77-1.41 | 0.79 | - | - | - |
| Antiplatelets | 0.98 | 0.73-1.33 | 0.91 | - | - | - |
| Beta-blockers | 0.75 | 0.55-1.02 | 0.07 | - | - | - |
| ACEi/ARB-II | 0.99 | 0.65-1.51 | 0.97 | - | - | - |
| Spironolactone | 1.59 | 1.15-2.20 | 0.005 | 2.07 | 1.42-3.03 | <0.001 |
| Loop diuretic | 2.82 | 1.79-4.46 | <0.001 | 2.00 | 1.20-3.27 | 0.008 |
| Haemoglobin | 0.99 | 0.98-1.00 | 0.009 | - | - | - |
| Red cell count | 0.81 | 0.62-1.07 | 0.14 | - | - | - |
| Mean corpuscular volume | 0.98 | 0.96-1.00 | 0.06 | - | - | - |
| Red Cell Distribution Width* | 1.33 | 1.24-1.43 | <0.001 | 1.23 | 1.13-1.33 | <0.001 |
| White Blood Cell count* | 0.98 | 0.93-1.04 | 0.57 | - | - | - |
| Neutrophiles (%) | 1.02 | 1.01-1.04 | 0.007 | - | - | - |
| Lymphocytes (%) | 0.97 | 0.95-0.99 | 0.001 | - | - | - |
| Neutrophile/Lymphocyte ratio | 1.04 | 1.01-1.07 | 0.01 | - | - | - |
| Platelets* | 0.99 | 0.99-1.00 | 0.004 | 0.99 | 0.99-1.00 | <0.001 |
| Mean Platelet volume | 1.03 | 1.01-1.04 | 0.002 | - | - | - |

Abbreviations. NYHA: New York Heart Association; ACE-I: angiotensin converting enzyme inhibitors; ARB-II: Angiotensin II receptor blockers; CRT-D; cardiac resyncronization therapy-defibrillator; CRT: cardiac resynchronization therapy; eGFR: estimated glomerular filtration rate; CM: cardiomyopathy; LVEF: left ventricular ejection fraction; COPD: chronic obstructive pulmonary disease; AF: atrial fibrillation; AAA: abdominal aortic aneurysm; HR: hazard ratio; CI: confidence of interval

*RDW (per % increase); *platelets (per % increase); *white cell count (per % increase)

**Brief Summary**

Almost a third of patients have suboptimal responses following cardiac resynchronization therapy (CRT). This is the first powered single-centre study to demonstrate that red cell distribution width and platelet count are independent predictors of long-term all-cause mortality in CRT patients.
